# Experimental Evolution in Plant-Microbe Systems: A Tool for Deciphering the Functioning and Evolution of Plant-Associated Microbial Communities

**DOI:** 10.3389/fmicb.2021.619122

**Published:** 2021-05-07

**Authors:** Beatriz Manriquez, Daniel Muller, Claire Prigent-Combaret

**Affiliations:** UMR 5557 Ecologie Microbienne, VetAgro Sup, CNRS, INRAE, University of Lyon, Université Claude Bernard Lyon 1, Villeurbanne, France

**Keywords:** experimental evolution, synthetic community, interaction network, microbiota, holobiont, evolutionary adaptation

## Abstract

In natural environments, microbial communities must constantly adapt to stressful environmental conditions. The genetic and phenotypic mechanisms underlying the adaptive response of microbial communities to new (and often complex) environments can be tackled with a combination of experimental evolution and next generation sequencing. This combination allows to analyse the real-time evolution of microbial populations in response to imposed environmental factors or during the interaction with a host, by screening for phenotypic and genotypic changes over a multitude of identical experimental cycles. Experimental evolution (EE) coupled with comparative genomics has indeed facilitated the monitoring of bacterial genetic evolution and the understanding of adaptive evolution processes. Basically, EE studies had long been done on single strains, allowing to reveal the dynamics and genetic targets of natural selection and to uncover the correlation between genetic and phenotypic adaptive changes. However, species are always evolving in relation with other species and have to adapt not only to the environment itself but also to the biotic environment dynamically shaped by the other species. Nowadays, there is a growing interest to apply EE on microbial communities evolving under natural environments. In this paper, we provide a non-exhaustive review of microbial EE studies done with systems of increasing complexity (from single species, to synthetic communities and natural communities) and with a particular focus on studies between plants and plant-associated microorganisms. We highlight some of the mechanisms controlling the functioning of microbial species and their adaptive responses to environment changes and emphasize the importance of considering bacterial communities and complex environments in EE studies.

## Introduction

In nature, microorganisms are living inside complex microbial communities (i.e., microbiota) where they evolve under constant interaction with sympatric microbial populations (Brockhurst et al., 2003; Brockhurst and Koskella, 2013; Guan et al., 2013). While tremendous progress has been achieved regarding the description of natural microbial community composition, understanding their functioning, their structure dynamics and how they may evolve is still in its infancy (Rainey and Quistad, 2020). Myriads of interactions (e.g., cooperation, competition, or predation) occur between microbial community members which are influenced by all the biotic and abiotic factors the microbial community faced up. In turn, the functioning of microbial communities affects their environment leading to further changes in biotic interactions and in evolutionary dynamics of its members. Microbial communities are thus dynamical systems.

The environmental persistence and functioning of microbial communities are influenced not only by their species richness but also by their functional diversity. There are thus two important aspects of microbial communities: their taxonomic structure (diversity and abundance of each individual population within the community) and how they function (community behavior and activities) (Little et al., 2008). Microbial communities are composed of various functional groups which could be defined as all populations doing the same function (Bouffaud et al., 2016). The same population could belong to several functional groups, which leads to interconnected functional networks within a single microbial community (Bruto et al., 2014; Renoud et al., 2020). Reconstructing the structure of functional and metabolic networks within microbial communities and understanding how this structure might evolve over time is largely unknown even when imposing stable laboratory environmental conditions. However, there is a great expectation placed on experimental evolution (EE) studies to unravel the evolutionary dynamics of populations within microbial community, although EE studies on natural microbial community are so far uncommon.

Experimental evolution corresponds to the study of the evolutionary modifications occurring on populations in response to environmental conditions imposed by the experimenter (Kawecki et al., 2012). It made it possible to monitor the real-time adaptation of populations to their environment (biotic and abiotic) by observing evolution in real-time for ten, hundreds or even thousands of generations and detecting phenotypic or genetic changes between individuals in the populations. The use of experimental replicates allows to disentangle the contribution of chance events (such as drift and founder effects) and the contribution of the imposed selective pressure. Thus, unlike carrying out genomic analysis on existing natural organisms and then interpreting their evolution, EE makes it possible to transform evolutionary genetics into a prospective undertaking, and to decipher the genetic bases of adaptation (Van den Bergh et al., 2018).

The first experiments with continuous-cultures were employed in the early 1950s with microorganisms and focused on describing dominant mutant phenotypes favored during extended growth (reviewed in Adams and Rosenzweig, 2014). In the decades that followed, scientists attempted to correlate the observed phenotypic changes with gene mutations or duplications, revealing that genetic adaptation is the basis of evolutionary adaptation. Genetic changes happen rapidly, within the first hundred cell generations, for a bacterial monoculture grown in a unique and limited resource environment (Lenski and Travisano, 1994; Cooper and Lenski, 2000; Wiser et al., 2013; Kram et al., 2017; Lenski, 2017). Bacteria are powerful candidates for EE as they offer short generation time and large population size, so multiple mutations can be present simultaneously. Moreover, bacteria are easy to track (i.e., isolation, numeration) and store, this allows to compare competitive fitness between evolved and ancestral genotypes. A regular collection and conservation of samples during EE and further genomic, genetic and/or phenotypic analyses could be done to decipher the evolution process (Jerison and Desai, 2015; Marchetti et al., 2017). Indeed, genomic and molecular data are currently available for many bacterial species, as well as techniques for their precise genetic analysis and manipulation (Vacheron et al., 2018; Batstone et al., 2020; Li et al., 2020; Scheuerl et al., 2020; Rodríguez-Verdugo and Ackermann, 2021).

**FIGURE 1:**
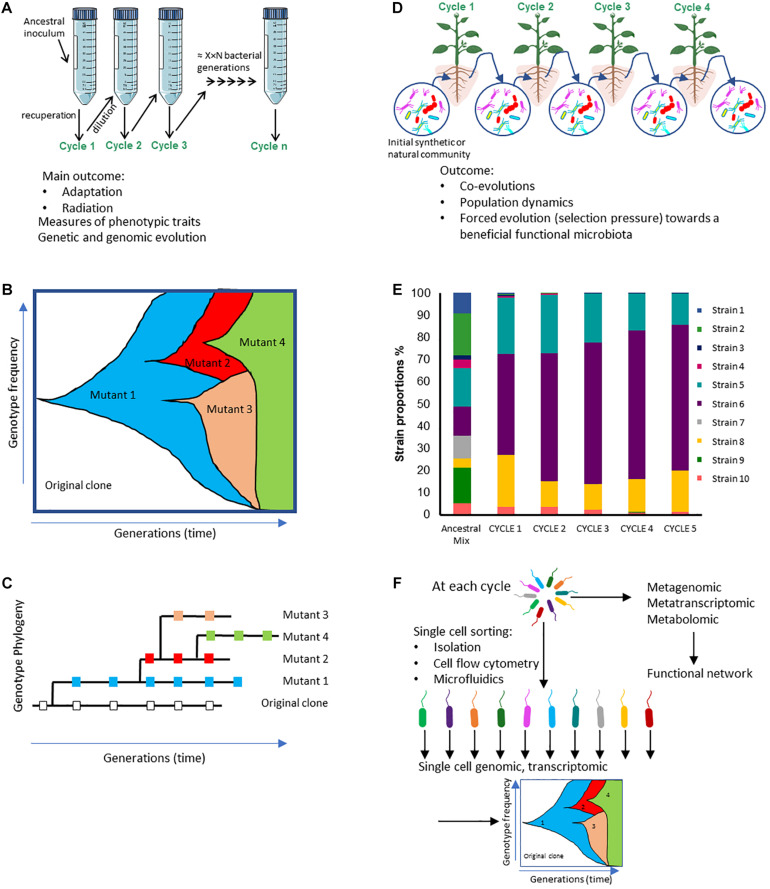
Comparison of the potential outcomes from experimental evolution (EE) studies starting with **(A)** a single clone or **(D)** a microbial community. **(A)** Example of an experimental evolutionary design based on a single clone serially cultivated in a simple medium, a stressful or a changing environment; those experiments enable to track phenotypic and genetic changes over time. **(B)** Theoretical Muller diagram depicting the distinct genotypes frequencies over microbial generations that could be observed in EE study described in A. **(C)** Theoretical phylogenetic tree built from genotypes that can be sampled from different time points throughout the EE described in A and sampled at the same generation times. **(D)** Example of an experimental evolutionary design based on an inoculum made up of several microorganisms (synthetic or natural communities); those experiments make it possible to monitor changes in population levels **(E)**, and genetic, transcriptomic or metabolomic changes over time **(F)**. **(E)** Theoretical bar graph illustrating the composition of a microbial community showing differences in taxa among both the inoculum (ancestral mix) and different experimental cycles. **(F)** At each cycle, approaches could be implemented at the scale of the whole community (i.e., meta-omics) or of individual cells after cell-sorting (single-cell sequencing, transcriptomic).

Nowadays, mass sequencing allows to unravel the genetic mechanisms underlying adaptation in bacteria at the genomic level (Kawecki et al., 2012; Bailey and Bataillon, 2016). EE can now be used to answer more complex ecological questions, such as evolutionary adaptation during biotic interactions between a bacterial population and a host or between bacterial populations within synthetic or natural microbial communities (Brockhurst and Koskella, 2013; Table 1 and Figure 1). It is also a powerful tool to decipher the underlying mechanisms of virus-bacteria co-evolution (Paterson et al., 2010; Scanlan et al., 2011), adaptation of pathogens to humans (Yang et al., 2011) or evolution of animal gut microbiota (Lescat et al., 2017; Tso et al., 2018) but the latter topics are not discussed in the present review. Here, a particular focus was given, but not limited, to EE studies done with plant-microbe systems. We compare systems with increasing complexity (Table 1). First, using selected examples, we describe EE studies involving a single microbial species evolving under low complexity environmental conditions. These studies allowed to infer the genomic mechanisms underlying bacterial adaptation to new environments. Second, we analyse EE studies using more complex systems, such as those investigating the interaction between single microorganism and the plant. These EE studies shed light on the role of the eco-evolutionary feedbacks during microbe-plant interactions. Third, we examine the importance of considering synthetic or natural bacterial communities (or microbiota) and complex environments in EE studies. Finally, we discuss future avenues of EE studies and point out the gaps must be bridged to analyze complex systems with the same detailed analysis of genomics adaptation than simple systems (Figure 1).

**TABLE 1:** Selected examples of experimental evolution studies (with a focus on interactions with plants at the microcosm scale), that have contributed to advances in genomic evolution, horizontal gene transfer, and plant host–microbe interactions.

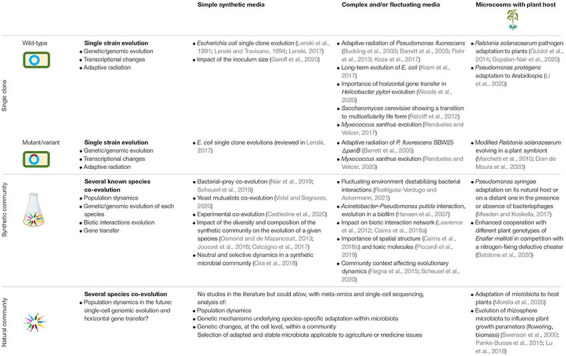

## Experimental Evolution to Decipher the Adaptive Evolution of Single Microbial Population in Synthetic Systems of Low Complexity

Experimental evolution has mostly been carried out on very simple conditions: using a single organism evolving in a low complexity system (Barrick et al., 2009). These EE experiments have provided essential knowledge on the molecular evolution and adaptive changes of microorganisms, particularly bacteria, to experimenter-imposed conditions and allowed to uncover the correlation between genetic and phenotypic changes (Figure 1).

### Bacterial Adaption and Key Evolutionary Driving Forces

Adaptation of populations mostly happens through mutations that can improve the performance of organisms in their environment and therefore improve their fitness (Lang et al., 2011). Distinct environmental conditions will impose different selection pressures and will determine whether or not a mutation would be beneficial or detrimental in a given niche at a given time. If a beneficial mutation happens on a single clone it will need a certain amount of time to rise to the population level (fixation). According to Fisher’s model, the time required for a mutation to fix is inversely proportional to its beneficial effect (Fisher, 1930; Tenaillon, 2014). However, stochastic extinction of a mutation, even if it may confer some fitness advantages, or increase in frequency of a deleterious mutation can exist. So called “genetic drift” also plays an important role in fixation of mutation in a population and represents the role of chance in evolution (Kayser et al., 2018; McDonald, 2019). The spatial distribution of cells will also influence the genetic drift’s force and competition between mutated clones (Kayser et al., 2018). However, bacteria reproduce asexually, so the different genotypes that rise in the population do not recombine and the corresponding mutations they harbor may not reach fixation within the population. This phenomenon is known as clonal interference and influences the dynamics of evolution and adaptation as clones with beneficial mutations will interfere with each other’s spread in the population (Gerrish and Lenski, 1998; McDonald, 2019). However, motility and dispersal may help competitive populations to coexist in a same system through active segregation and spatial exclusion (Gude et al., 2020).

### Parallel and Convergent Evolution of Single Microbial Species in Simple Environments

Given that evolution in bacteria happens mostly by random mutations, an important question arises from EE studies: are they repeatable? Do same genotypic and phenotypic changes arise in an evolved microbial population submitted to same environmental changes? In order to better understand evolutionary adaptation, genotype-to-phenotype correlations need to be more fully investigated. This can be done by focusing on patterns of parallel and convergent evolution.

Parallel evolution is defined as the independent evolution of similar phenotypic traits in lineages closely related to each other and involving changes in orthologous genes, whereas convergent evolution concerns non-related phylogenetic lineages and changes in non-homologous genes (Bai et al., 2015; Pickersgill, 2018).

Many EE studies have shown that a single clonal ancestor can give rise to independently evolved populations sharing similar traits under the same environmental conditions (Schluter et al., 2004; Colosimo, 2005; Zhang et al., 2005). Lenski’s experimental evolution study is composed of 12 replicate populations of *E. coli* B, which are currently still evolving on a glucose-limited minimal salts medium (Lenski et al., 1991; Lenski, 2017; Consuegra et al., 2021; Table 1). DNA microarray analyses of two evolved populations showed similar changes in the transcription of 59 genes after 20,000 generations; the genetic bases of these changes were investigated and relevant parallel mutations were found on many of the independently evolved populations (Cooper et al., 2003).

In simple organisms, biological functions are mostly encoded by single genes, whereas, in higher forms of life, more complex regulatory networks are involved. In bacteria and yeast, complex functions are often related to modules of genes (Hartwell et al., 1999). Reflecting this, parallel evolution will not always mean changes in the same genes but rather similar changes in related gene modules. Herring et al. (2006) explored the parallel changes in metabolic and regulatory networks that arose in five *E. coli* populations that evolved separately. They proved, combining mutant approach and whole-genome resequencing of five clones of *E. coli*, that 13 different spontaneous mutations were responsible for improved fitness during adaptation to a glycerol synthetic growth medium (Herring et al., 2006). This study provides a clear example of how different changes can have similar phenotypic effects (Table 1). Dissimilar genetic changes can lead to parallel phenotypes, meaning that functional connections within and between genetic modules can be established by pairing experimental evolution to whole genome sequencing (Segrè et al., 2006; Yeh et al., 2006).

However, clonal populations evolving in an identical environment could reach different fitness rates and may diverge (Lenski et al., 1991). This divergence reveals that there may be more than one adaptive strategies under the same environmental conditions (Elena and Lenski, 2003). On the other hand, we could expect that experiments ran with genetically different organisms led to different fitness. Travisano et al. (1995) evaluated fitness on maltose of several *E. coli* lines that had evolved in glucose for 2,000 generations. Lines that started with the lower fitness in maltose improved faster, but all the lines tend eventually to converge to a similar fitness on maltose evidencing convergent evolution.

### EE of Single Microbial Species in Variable and Complex Environments

In the past, EE studies were mostly done in simple environments, where the concentration of one essential source of carbon available to all individuals controls the population growth rate (Hillesland and Stahl, 2010; Yu et al., 2017).

This selects for different types of genotypes throughout experiment transfers. Heavy consumers and fast-growing microbes show increased fitness in the beginning of the experiment. When the resource starts to be depleted, genotypes that are able to survive with low resources and metabolic by-products increase in frequency. Finally, when there are almost no resources, there is an increase in prevalence of genotypes that are able to either metabolize toxic by-products (e.g., Blount et al., 2012) or are able to grow in very small amount of resources. This may lead to the evolution of two types of genotypes: generalist (able to consume a large range of the available resources) or specialist (able to grow faster than generalists, but in a shorter range of resources). This ensures the maintenance of polymorphism in population, with all genotypes present in the population, but with their prevalence varying until the cycle is started again with a new medium transfer. In complex environments that contain several resources, cell populations may become wide specialists able to consume various carbon substrates.

In the laboratory, because of the complexity of evolutionary processes, most of the EE assays have been carried out in a constant environment, using chemostats or continuous culture systems, so the growth conditions would remain the same for the whole experiment (Treves et al., 1998; Maharjan et al., 2006; Gresham and Hong, 2015). Recently, Westphal et al. (2018) used a batch culture system to study the impact of four changing environments on the adaptation of *E. coli*. In these systems there is no addition of nutrients, therefore the cultures experience fluctuation in nutrient availability. Therefore, as nutrients are consumed, waste products are released and less energetically favorable metabolisms become important for survival. Their data revealed that different environments may select different mutations; this emphasizes the importance of performing experimental evolution in complex and ever-changing environments. To take these findings one step further, Kram et al. (2017) carried out an EE assay with *E. coli* in a complex and variable environment (i.e., in a rich medium LB and with serial passages every 4 days). This scheme allows cells to go through all phases of growth and to adapt to different stresses (nutrient limitation, oxidative stress and pH variation). This experiment showed that after only 30 generations, evolved populations presented changes in growth rates but also adaptive mutations allowing the cells to cope with the varying stresses arising during the culture (Table 1). They also evidenced parallel changes in evolved populations (Kram et al., 2017).

The importance of environmental complexity and evolution of niche width have been studied by Barrett et al. (2005) who compared the evolution of more than one hundred replicate lines of the bacterium *Pseudomonas fluorescens*, over ∼900 generations in 15 environments of different complexity (Table 1). To do this, the authors used a synthetic growth medium that contained 1–8 single carbon substrates or specific combinations between them, and compared the genetic evolution of *P. fluorescens* lineages in simple and complex environmental growth media. In complex medium, the *Pseudomonas* lines evolved into several co-existing genotypes (adaptive radiation), exhibiting greater fitness for a wider range of carbon sources (but not for all), than the lines that evolved in simple environments. A higher fitness variance within populations selected in complex environments was thus observed. Indeed, lineages evolved in simple environment specialized in consuming a single carbon substrate, while those evolved in complex media were able to consume several substrates (but not all), without any appreciable loss of functions or apparent fitness costs. These results suggest that evolution in complex environments will lead to the emergence of imperfect generalist overlapping lines, adapted to a certain range of substrates but not to all (Barrett et al., 2005).

**FIGURE 2:**
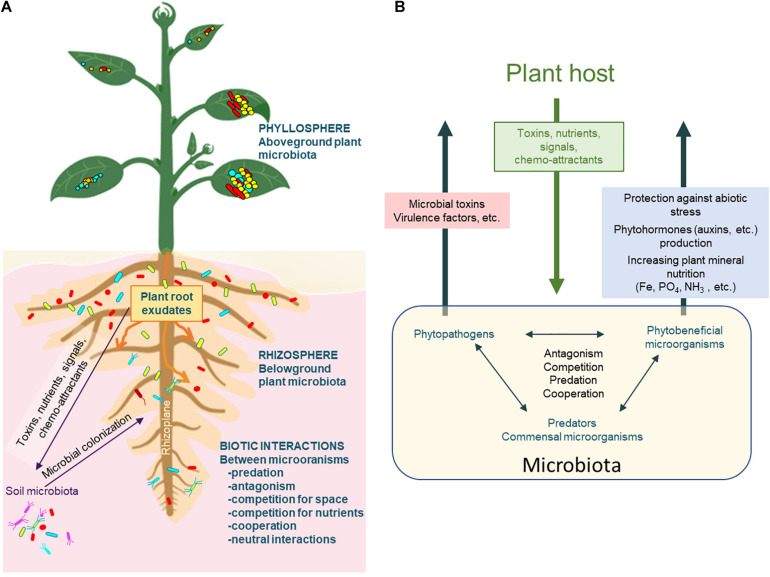
Relationships between plant and microbial components. **(A)** Plants interact with a wide diversity of microorganisms (microbiota) in the different compartments of the plant system. The rhizosphere corresponds to the soil zone where the roots impact the soil organization and microbial functioning, while the rhizoplane is the interface between the root surface and the soil. The phyllosphere corresponds to the surface of all the aerial organs of the plant. Root exudates attract or repel soil microorganisms toward roots and increase the growth of myriads of microorganisms that will interact between each other (positive, negative or neutral biotic interactions). **(B)** Major biotic interactions in the rhizosphere include plant-microorganism interactions and microorganism/microorganism interactions, involving the exchange of different classes of molecules (hormones, toxins, virulence factors, signals, etc.).

In nature, communities grow under conditions where many substrates are available, supporting a great number of consumer strategies for microorganisms. However, the availability of these substrates can be heterogeneous (both in space and time), leading to fluctuating selection for different genotypes within microbial populations, or to varying species within the microbial community. The presence of biotic interactions can also be a cause of increased environmental complexity (Brockhurst and Koskella, 2013). However, this environmental component was largely overlooked until more recently. A great example of how biotic interactions provide spatial and temporal heterogeneity is the plant rhizosphere (Kuzyakov and Razavi, 2019; Figure 2 and Box 1).

**Box 1.**

The plant rhizosphere, a rich and heterogeneous environment. Many soil microorganisms are capable of colonizing the plant rhizosphere, the volume of soil under the influence of root activities. In the rhizosphere plants exert a selection on soil microorganisms by secreting around their roots a wide range of compounds known as plant exudates (Figure 2). About 5–21% of the total carbon fixed by photosynthesis has been reported to be exuded at the root level and to influence the composition of the rhizosphere microbiota (Derrien et al., 2004; Vandenkoornhuyse et al., 2015). The exudates contain, among other things, nutrients but also signaling and chemo-attracting molecules. Along the root system, the exuded molecules are not evenly distributed. This leads to a heterogeneous root and rhizoplane colonization by selected microorganisms able to use the nutrients locally available. Some of these microorganisms will cooperate with the host plant through diverse mechanisms, like increasing the plant’s mineral and nitrogen nutrition, synthesizing phytohormones influencing plant phenotypes or stimulating the induced systemic response (Vacheron et al., 2013; Figure 2). The nature of the exudates will impact the composition and functions of the root-associated microbiota which in turn will affect the physiology of the plant, generating new exudates (Vacheron et al., 2013; Chaparro et al., 2014). Thus, the rhizosphere is a discontinuous environment both in space and time, rich in nutrients, locally increasing the growth of highly diverse microbial populations that are ultimately in constant competition for nutrients. These competitive conditions make the rhizosphere a hotspot for horizontal gene transfer (HGT, van Elsas et al., 2003), mutations or gene arrangements, and will drive bacterial adaptation and acquisition of new genes (Wisniewski-Dyé et al., 2011). The latter will result in population genotype and phenotype changes, in the modification of the structure and function of the community and hence, will have profound effects on the long-term evolution of communities. Nevertheless, understanding how rhizosphere community members may evolve, at a species, sub-species or even individual cell level, is still a complex issue. Right now, rhizosphere natural communities and plant rhizosphere systems have rarely been included in EE studies. But, thanks to the development of single cells sequencing, considering such complex and heterogeneous living system in EE studies is becoming feasible and will allow to obtain a comprehensive detailed view of rhizosphere communities functioning and driving forces (Figure 1).

## Experimental Evolution With Complex Plant-Microbe Systems

Plant select microorganisms in the rhizosphere through their exudates (Figure 2). Interactions between plant and microorganisms can be beneficial, neutral or deleterious and several studies have used EE to unravel the relationships between the plant and plant-associated microorganisms (Table 1). Here we review how these EE assays have helped to better understand plant-microorganism’s interactions, first for plant pathogens and secondly for plant-beneficial bacteria.

### Plant Pathogens

Plant pathogens are pervasive, and their management is important for agriculture and food supplies, with direct impacts in human health and welfare. Thus, understanding the mechanisms underlying host co-evolution can help on devising new strategies to eradicate these types of pathogens. It is known that pathogen and plant defenses co-evolve, which translates into an arms race, where host and pathogen constantly evolve by mirroring the response of one another (Brockhurst and Koskella, 2013). Deciphering the genetic bases of pathogen adaptation is thus critical to understand disease emergence and acquisition of novel traits by pathogens when colonizing hosts (Toft and Andersson, 2010; Gandon et al., 2013).

EE approaches have been widely used to study the ecology of plant pathogens and dynamics of their adaptation when they interact with new host. Here, a focus is made on *in planta* EE experiments done with bacterial plant pathogens (Table 1), but others studies have also focused on fungal (Gilbert and Parker, 2010) or virus plant pathogens (Bedhomme et al., 2012).

Indeed, in an attempt to experimentally study the genetic basis of adaptation to new host, Guidot et al. (2014) performed EE with *Ralstonia solanacearum*, a plant pathogen with a continuously broadening host spectrum. A single clone of the model strain GMI1000 was inoculated on three native host plants (tomato, eggplant and pelargonium) where the pathogen causes disease and two distant plants (cabbage and bean) where it grows asymptomatically. The pathogen was transferred serially to the same plant line, in order to maintain the pathogen on the same host for 300 bacterial generations (26 serial passages). Although evolved strains showed an increase in competitiveness (pathogenicity) in both host and non-host plants, this increase was greater in the non-host plant (cabbage and beans), to which the pathogen was originally not adapted. This rapid evolution when colonizing a distant host plant tends then to decrease until reaching an optimum. This is known as “diminishing returns epistasis” that is often observed during EE studies with almost invariantly a reduction of adaptation speed and of mutations’ fitness gain during the adaptation (Couce and Tenaillon, 2015).

Whole-genome analysis and comparison of the ancestral GMI1000 strain with nine evolved clones (three from the tomato host and six from the bean) highlighted that only few genes contribute to adaptation to a specific host. In particular, the transcriptional regulator encoding gene *efpR* was identified as important for the adaptation of *R. solanacearum* to bean. Evolved clones harboring *efpR* mutations had a greater competitiveness compared to the wild type clone during co-infection of bean plants (Guidot et al., 2014). EfpR was thereafter identified as a global catabolic repressor and regulator of virulence traits, whose mutations allow an enhanced metabolic versatility and adaptation to host vascular tissues of new hosts (Perrier et al., 2016). This study highlighted the importance of combining EE with whole-genome sequencing in order to unravel the genetic basis of pathogen adaptation. More recently, transcriptomic analyses combined with genomic sequencing evidenced that epigenetic modifications also occur during the adaptive evolution of *R. solanacearum* to a tomato resistant cultivar, allowing expression changes of the EfpR regulon (Gopalan-Nair et al., 2020).

Another EE study focused on understanding how the prior evolutionary history of a pathogen affected subsequent evolution in a new host. To this end, *Pseudomonas syringae*, a well-known plant pathogen, has been used to infect a distant host (*Arabidopsis*) or a native host (tomato) over several infection cycles, in the presence or absence of phages. Bacteriophages impose evolutionary trade-offs on the bacteria they infect, triggering modifications of bacterial surface receptors that subsequently may impact *P. syringae* interactions with distinct hosts. Selection on *Arabidopsis* (through serial passaging) leads to a larger increase in pathogen growth rate on both hosts, than selection on tomato. These results point out that a given association between a plant and a pathogen may affect the growth rate of the pathogen on other plants it will subsequently infect (Meaden and Koskella, 2017).

Other EE studies done with *Agrobacterium* have highlighted the appearance of cheaters in pathogen populations that will benefit of the production of virulence factors by the rest of the population without producing them themselves (Tannières et al., 2017). Indeed, the production and regulation of virulence factors are usually associated with high costs (energy or fitness) and are finely regulated by quorum sensing signals in *Agrobacterium*. Tannières et al. (2017) showed that cheater mutants that minimize the costs of expressing quorum sensing regulated functions spread during EE.

### Non-pathogenic Plant Associated Microorganisms

EE studies have also investigated the genetic mechanisms underlying adaptation of bacteria that present mutualistic or synergistic plant-microbe interactions.

*Fabaceae*-rhizobium interactions are known as highly specific interactions between a bacterial symbiont and its host plant. In this case, the plant rewards cooperative symbionts in detriment of less mutualistic microbes but without necessarily punishing the latter (Batstone et al., 2017). Using a year-long EE experiment between *Ensifer meliloti* and *Medicago truncatula*, Batstone et al. (2020) have shown that local and recent adaptation of the symbiont to a plant genotype increases cooperation, independently of host selection.

Another EE experiment has investigated which bacterial genes facilitate symbiosis between bacteria and plant using a legume symbiont and a non-symbiotic bacterium. To do this, *R. solanacearum*, hosting the *Cupriavidus taiwanensis* symbiotic plasmid of *Mimosa pudica*, was repeatedly inoculated on the plant host (Marchetti et al., 2010; Table 1). After a series of *Mimosa pudica* infection cycles, an evolved symbiont well adapted to the host (i.e., able to induce nodulation and infect nodules, however, not to fix nitrogen) was obtained. Sequencing of intermediate and final forms revealed that the symbiosis-adaptive mutations happened in global regulatory proteins, leading to a reworking of the regulatory systems in *R. solanacearum*. These adaptive mutations included the inactivation of the type III secretion systems (the main virulence factor of *R. solanacearum*; Genin and Denny, 2012) and modifications on the expression of *efpR* (Guan et al., 2013; Capela et al., 2017). Genomic analyses revealed that is not only the *efpR* gene that is mutated but also its upstream region. Altogether these mutations led to metabolic and transcriptomic changes, allowing mutualistic interaction with the plant (Capela et al., 2017; Marchetti et al., 2017; Doin de Moura et al., 2020). These findings highlight how EE can help to identify the evolutionary pathways that drive the evolution of symbiotic functions in rhizobia, by understanding how adaptation modifies the regulatory systems that control virulence and determine the ecological functions of bacteria.

Other plant-beneficial bacteria are involved in less specific interactions with plant. These bacteria known as Plant Growth-Promoting Rhizobacteria (PGPR) colonize the plant rhizosphere (Figure 2). EE assays carried out *in planta* with plant-inoculated PGPR are uncommon. To our knowledge there is only one open resource work dealing with EE during plant-PGPR interaction (Li et al., 2020). In this work, a well-known biocontrol PGPR *Pseudomonas protegens* CHA0 was grown on the roots of *Arabidopsis thaliana* (on five independent Col-0 replicates) in gnotobiotic conditions, during 6 months (i.e., six one-month plant growth cycles). The bacterium was shown to evolve to a more mutualistic relationships with its plant, acquiring an improved competitiveness for root exudates, a better ability to tolerate plant-secreted antimicrobial compounds and a stronger positive effect on the plant performance. Different mutations in the key two-component regulator system GacS/GacA were recorded, conferring higher competitiveness to evolved CHA0 clones compared to the ancestral form in the presence of *A. thaliana* plants.

## Experimental Evolution Studies With Synthetic and Natural Microbial Communities

A large portion of studies on EE and microbial adaptation focuses on one single species, considering the genetic diversity within an evolving population rather than diversity between species within communities (e.g., Wiser et al., 2013; Kram et al., 2017; Lenski, 2017; Garoff et al., 2020). However, microorganisms are indeed in constant interaction with other organisms in their environment, so that any population in nature evolves with the other sympatric microbial populations.

Species interactions can influence how species evolve and adapt to environmental changes (Gorter et al., 2020). Unraveling the interactions that take place within a community is essential to understand how a community carries a function and how it will respond to perturbations and may evolve. For example, within a microbial community, some species can use the waste products generated by others (Lawrence et al., 2012; Seth and Taga, 2014; Piccardi et al., 2019). These interactions can become so relevant that a given microorganism can present a lower growth rate or even may not grow when cultivated alone, compared to when cultivated with another microorganism (Hillesland and Stahl, 2010) or within a community (Smith and Schuster, 2019). However, adaptation of one population to a new environment could be favored or counteracted by sympatric populations (Castledine et al., 2020). This process of reciprocal adaptation by interacting species is defined as co-evolution (Buckling and Rainey, 2002). Sympatric populations could be locally maladapted and may show trait convergence for the use of same resource and these competitive interactions are specific to the co-evolved community members (Castledine et al., 2020).

### EE With Synthetic Community in Environments of Increasing Complexity

Evolving together creates adaptive co-evolutionary dependencies: “it takes all the running you can do to stay in the same place,” meaning that species have to constantly adapt to the other evolving species in order to survive/maintain their fitness (i.e., “Red Queen hypothesis”; Van Valen, 1977; Strotz et al., 2018). Species that co-evolved increased competitive interactions between them. Coevolution between competitors is expected to change species abundances within the community and affect subsequent community evolution (Nair et al., 2019; Scheuerl et al., 2019, 2020; Figure 1).

Thus, co-evolution experiments focused initially on microorganisms sharing negative interactions (i.e., predation, antagonism) (Nair et al., 2019; Scheuerl et al., 2019). Nowadays, co-evolution experiments also focus on mutualistic interactions, because these interactions are widespread in nature (Chomicki et al., 2020).

Several EE studies investigate the evolution of pairwise interactions between organisms (Nair et al., 2019; Scheuerl et al., 2019; Vidal and Segraves, 2020; Rodríguez-Verdugo and Ackermann, 2021). Here again, EE can be performed in constant environment (controlled microcosms with stable nutritive and physicochemical parameters), or in a fluctuating or complex environment (Table 1). Changes in the environment strongly constrain the existing biotic interactions between microorganisms. Rodríguez-Verdugo and Ackermann (2021) compared the evolution of co-cultures of *Acinetobacter johnsonii* and *Pseudomonas putida* in constant environment (the same nutrient medium at each cycle) to EE conducted in fluctuating environment (alternation of nutrient sources between each cycle). They showed that, the two species coexisted over 200 generations in the constant environment, whereas in the fluctuating environment, the extinction of one of the two partners was observed in half of the repetition, suggesting that the fluctuating environment destabilizes positive pairwise interactions.

However, natural microbiota are generally more complex and host a multitude of species of microorganisms, that will directly impact the evolutionary trajectory of each population (Jousset et al., 2016; Cira et al., 2018; Hall et al., 2020; Scheuerl et al., 2020), for example by suppressing competitors (Osmond and de Mazancourt, 2013), or by generating new niches (Calcagno et al., 2017). Species have thus to adapt not only to the environment itself but also to the biotic environment dynamically shaped by the other species (Ratzke and Gore, 2018; Scheuerl et al., 2020). Sharing the same ecological niche implies sharing some resources and this inevitably promotes competition within the group. Synthetic community approaches aim to mimic natural microbiota (Vorholt et al., 2017) and can help to better understand the functioning of biotic interaction network within natural communities (Figure 1). Cairns et al. (2018a) serially transferred a synthetic community of 33 bacterial strains on a complex liquid media. Over half of the strains from different species were lost in 16 days, after which the evolved community was relatively undisturbed until the end of the experiment (48 days). Within the evolved community, 14 strains co-existed with the predominance of three strains. The evolved synthetic community shared high diversity at different levels (i.e., taxonomic, metabolic, and functional levels) (Table 1).

Adaption to other competitive species may imply the production of antimicrobial metabolites, but a trade-off can arise due to the substantial energy-cost of their production (Yan et al., 2018). Some compounds can be costly to produce for one individual, but beneficial for all the members of the community. Microbes have thus developed multicellular cooperative behaviors, like biofilm formation and quorum sensing, along with nutrition acquisition, and the outcome of these interactions are referred as public goods (Besset-Manzoni et al., 2018; Smith and Schuster, 2019). Public goods take many forms from large proteins to small metabolites and can be actively or passively secreted, but one of their main features is that their benefit increases with population density.

Various EE studies comparing synthetic microbiota whose complexity increases (diversity and/or richness), increasing also the complexity of biotic interactions (competition for resources, cooperation, etc.), show that species adaptation largely depends on the community in which these species co-evolve. Thus, factors such as diversity or richness must be considered in microbial adaptation and evolution (Fiegna et al., 2015; Scheuerl et al., 2020). Another factor to consider in microbiota evolution is the impact of harsh conditions which may stimulate competition or cooperation. In a EE performed with synthetic microbiota, Scheuerl et al. (2020) adapted a continuous culture protocol and replaced the fresh medium addition at the end of each evolutionary cycle with the addition of only 10% of fresh medium, mimicking more natural conditions, and inducing competition and adaptation to recalcitrant carbon sources. This EE study revealed that the adaptation of bacteria to new environment is influenced by interspecies interactions.

Experimental evolution studies also enable to investigate the outcomes of interspecific interactions. For example, it is expected that plant root microbiota will be subjected to large environmental changes during plant development, which may lead to subsequent adaptation of the microbial community (Box 1). Specifically, a large number of natural compounds are exuded in the soil surrounding the roots, especially near the young parts, which modifies the physio-chemistry of the soil (oxygenation, pH, etc.), generating a stressful environment for the microbiota. Such stressful environment can result in competition or cooperation between members of the community. Using a synthetic community composed of four bacterial species, Piccardi et al. (2019) showed that the interactions between species evolved differently if the environmental stress level is low or high (stress gradient). Indeed, in a mild-stress environment, species evolved competitive interactions whereas, in harsh and toxic conditions, members of the community evolved cooperation or neutral behavior.

Unfortunately, cooperative behavior is not the only evolutionarily stable strategy as cheaters may appear and invade the community. Cheaters are non-cooperative individuals that benefit from the public goods without producing them. By not paying the cost of their production, cheaters could have more energy allocated to their growth, and therefore their relative fitness increases. The underlying mechanisms of cheaters’ loss of function often involve a selective gene loss to optimize their adaptation to the environment (i.e., the “Black Queen Hypothesis,” Mas et al., 2016). This evolutionary strategy has great impact on long term interactions because the fitness of cheaters depends on the public goods provided by the cooperating microbes. The stability of the community can only be maintained if the proportion of cheaters remains low within the community. Ultimately, cheaters will reduce the effective population size of the cooperating microbes, reducing the rate of public goods production, but also the rate at which beneficial mutations would arise in the community and the species would be able to adapt to a novel environment. The evolution of cooperation is a tricky issue since all microorganisms will tend, according to the natural selection theory, to maximize their own fitness. However, cooperative behavior is ubiquitous even though selfish interests have always been a source of conflict (Sachs and Wilcox, 2006; Burt and Trivers, 2009; Oliveira et al., 2014).

Batstone et al. (2020) investigated the prevalence of cheaters and non-cheaters in a simplified plant root community by performing EE with two strains of *Ensifer meliloti*. Both of these strains were able to receive carbon from the plant, but one strain lost the ability to fix N_2_ to feed back to the plant (cheater), and another strain maintained that function (cooperative). After 1 year, the authors observed that the frequency of these two strains varied according to the level of coevolution with the plant. On one hand, in the initial stages of the EE, the cheater presented a twofold fitness advantage in host colonization, but by the end of the experiment it was extinct in the five plant genotypes tested. On the other hand, the N_2_-fixing bacteria, which are less efficient in the first cycles of EE, became dominant at the end of EE. The interaction of evolved clones with the five *Medicago* lines showed that evolved clones achieved a higher fitness and provided greater benefits on the genotype with which they shared evolutionary history. Overall, this study suggests that cheaters are not able to outcompete cooperative genotypes, once they co-evolved with their host (Batstone et al., 2020).

Despite these recent studies, we still lack information on the eco-evolutionary dynamics behind cooperative and mutualistic behaviors. Moreover, deeper insight into biotic interactions within microbial communities is thus crucial to understand adaptation to novel environments. It can help in deciphering the dynamics of living systems and to predict responses to anthropogenic changes in the natural environment (Winder and Schindler, 2004; Davis et al., 2005; Berg et al., 2010).

### EE With Natural Microbial Communities in Complex Environments

Numerous studies have taken an interest in plant-associated microbiota, illustrating the ability of the microorganisms to positively influence plant health and developmental traits such as disease resistance, herbivory, abiotic stress tolerance and growth (Mendes et al., 2011; Grunseich et al., 2020). During plant development, plant-microbiota interactions can change, which may in turn impact plant development (Swenson et al., 2000). For instance, an artificial microcosm selection carried on *A. thaliana* showed that plant biomass levels can be modified by soil microorganisms. Several *A. thaliana* seeds were inoculated with non-sterile soil batch and let grow for 35 days, which corresponds to one microcosm cycle. After each cycle, the plants presenting the highest and lowest biomasses were selected. The soil from these plants was retrieved and used to inoculate the next batch of plants. The experiment was carried for 16 microcosm cycles and both artificial selections (high or low biomass) were analyzed. *Arabidopsis* inoculated with the soil community from “high biomass” plants presented indeed higher biomasses than those inoculated with the soil community from “low biomass” plants. After 13 cycles, a soil analysis revealed different soil characteristics for high and low biomasses, that may reflect differences in the biotic components of the corresponding soils (Swenson et al., 2000; Figure 1).

Plant flowering is another trait that can be modulated by plant-associated microorganisms and the contributing microbiota’s populations can be experimentally selected and enriched from one microcosm cycle to the next. In a similar way to Swenson and collaborators’ experiment, several microcosms of *A. thaliana* Col0 were created with seeds placed on sterile soil. In this experiment, microcosms were selected for either an early or late flowering phenotype. Plants were harvested and the soil retrieved as soon as all the plants of the microcosm flowered, therefore, the duration of the microcosm cycle depended on the flowering time. After ten cycles on *A. thaliana* Col0, microorganisms were retrieved and inoculated on other *A. thaliana* genotypes and on a related crucifer, *Brassica rapa*. Plant-associated microorganisms induced on these plants the same phenotype (early flowering) as the one induced on the genotype (Col0) used for selecting the evolved community (Panke-Buisse et al., 2015). Rather than imposing no selective pressure on the plant host, in these studies, researchers selected a particular plant trait (biomass or flowering) to select plant-associated microorganisms contributing to this plant trait (Table 1). In a similar approach, Lu et al. (2018) selected evolved microbiota capable of either stimulating precocious flowering or delaying it. By studying the molecular interactions between root exudates and the microbiota, a new network of molecular interactions was established, linking the production of auxin phytohormone (i.e., indole-3-acetic acid) from tryptophan by the microbiota, the nitrogen cycling and the timing of flowering in the host plant. EE on natural communities thus enabled to document novel metabolic networks in which soil microbiota influenced plant flowering time, thus shedding light on the key role of soil microbiota on plant functioning.

To better understand the holobiont’s mechanistic functioning, an EE study was recently done without taking into consideration improved plant health, growth or development phenotype as a selective pressure outcome of the evolutive process (Morella et al., 2020). The authors collected the leaf microbiota (phyllosphere) of field-grown tomato plants in order to spray it on fresh tomato plants. After 10 days of growth, the phyllosphere microbiota were sampled again, and used to inoculate a new round of plants. A total of four passages was done. They compare the impact of five genotypes differing in disease resistance genes in the selection of the tomato’s phyllosphere microbiota. They evidenced a strong selection of a stable microbiota adapted to this ecological niche, with a significant driven effect of the tomato genotype. Contrariwise to the initial leaf microbiota that was unstable, the evolved microbiota became well adapted to its host and robust to the invasion of the initial community (Morella et al., 2020).

In the rhizosphere, networks of within-microbiota interactions may also shape the evolution and stability of the community as a whole and the observed effects on the plant, affecting its development, health, and response to abiotic and biotic stresses (Figure 2). Deciphering the principles that underlying ecological and evolutionary properties of microbial communities can allow to build predictive models of ecological dynamics of microbial communities.

## Conclusion and Future Avenues for EE Studies

In natural conditions, microbes often coevolve within an interspecific community that may interact with other non-microbial organisms, such as plants. Understanding what drives all the different types of interactions with and within microbial communities, and, importantly, how they co-evolved, will allow to draw predictions on how microbial communities and their hosts respond to environmental changes.

EE assays have historically mostly focused on low complexity systems, allowing to understand the evolutionary adaptation of microorganisms to stressful conditions. EE studies combined with whole-genome sequencing have allowed us to understand the genetic bases separating an evolved clone from its original ancestor strain in a wide range of situations and to reveal the genetic and functional networks involved in microbial adaptation (Segrè et al., 2006; Bailey and Bataillon, 2016; Figure 1). To date, there have been very few EE studies done on more complex systems using natural microbial communities (Table 1). Indeed, whole genome sequencing of many individual clones from one population or from several populations evolving together is a big technological barrier to solve (Figure 1).

The combination of recent technological advances on meta-omic approaches, cell sorting and single-cell sequencing, will soon allow to investigate more deeply the genetic mechanisms underlying species-specific adaptation within microbial communities evolving in complex and heterogeneous environments, like the rhizosphere (Box 1 and Table 1). Genetic changes, random genetic drift and natural selection operate on each community member leading to the fixation of mutations, hence altering the genetic composition of populations and indirectly affecting species interactions that dictate community ecology. The intersection of ecology and evolution is key to understand microbial communities.

## Author Contributions

BM wrote the first version of the manuscript and figures. BM, DM, and CP-C corrected and improved the manuscript. All authors have read and agreed to the submitted version of the manuscript.

## Conflict of Interest

The authors declare that the research was conducted in the absence of any commercial or financial relationships that could be construed as a potential conflict of interest.
